# Functional Exhaustion of HBV-Specific CD8 T Cells Impedes PD-L1 Blockade Efficacy in Chronic HBV Infection

**DOI:** 10.3389/fimmu.2021.648420

**Published:** 2021-09-13

**Authors:** Sara Ferrando-Martinez, Angie Snell Bennett, Elisabete Lino, Adam J. Gehring, Jordan Feld, Harry L. A. Janssen, Scott H. Robbins

**Affiliations:** ^1^Microbial Sciences, Biopharmaceuticals R&D, AstraZeneca, Gaithersburg, MD, United States; ^2^Toronto Center for Liver Disease, Toronto General Hospital, University Health Network, Toronto, ON, Canada; ^3^Department of Immunology, University of Toronto, Toronto, ON, Canada; ^4^Late Stage Oncology Development, Oncology R&D, AstraZeneca, Gaithersburg, MD, United States

**Keywords:** chronic HBV infection, PD-L1 blockade, LAG3, exhaustion, HBV cure

## Abstract

**Background:**

A functional cure for chronic HBV could be achieved by boosting HBV-specific immunity. *In vitro* studies show that immunotherapy could be an effective strategy. However, these studies include strategies to enrich HBV-specific CD8 T cells, which could alter the expression of the anti-PD-1/anti-PD-L1 antibody targets. Our aim was to determine the efficacy of PD-L1 blockade *ex vivo*.

**Methods:**

HBV-specific CD8 T cells were characterized *ex vivo* by flow cytometry for the simultaneous analysis of six immune populations and 14 activating and inhibitory receptors. *Ex vivo* functionality was quantified by ELISpot and by combining peptide pool stimulation, dextramers and intracellular flow cytometry staining.

**Results:**

The functionality of HBV-specific CD8 T cells is associated with a higher frequency of cells with low exhaustion phenotype (LAG3^-^TIM3^-^PD-1^+^), independently of the clinical parameters. The accumulation of HBV-specific CD8 T cells with a functionally exhausted phenotype (LAG3^+^TIM3^+^PD-1^+^) is associated with lack of *ex vivo* functionality. PD-L1 blockade enhanced the HBV-specific CD8 T cell response only in patients with lower exhaustion levels, while response to PD-L1 blockade was abrogated in patients with higher frequencies of exhausted HBV-specific CD8 T cells.

**Conclusion:**

Higher levels of functionally exhausted HBV-specific CD8 T cells are associated with a lack of response that cannot be restored by blocking the PD-1:PD-L1 axis. This suggests that the clinical effectiveness of blocking the PD-1:PD-L1 axis as a monotherapy may be restricted. Combination strategies, potentially including the combination of anti-LAG-3 with other anti-iR antibodies, will likely be required to elicit a functional cure for patients with high levels of functionally exhausted HBV-specific CD8 T cells.

## Introduction

Public health awareness for chronic hepatitis B (HBV) infection have progressively increased in the last decade. Despite a preventive vaccine being available, coverage is still under 40% and prevention strategies are insufficiently implemented (1). More than 257 million people, a striking 3.5% of the worldwide population, are living with chronic HBV infection. Persistent viral replication and continuous liver necroinflammation eventually leads to cirrhosis, end-stage liver disease, hepatic decompensation and hepatocellular carcinoma (HCC) (2, 3) which, annually, results in more than 850,000 deaths (1). Current antiviral therapies (PEGylated-IFNα and nucleos(t)ide reverse transcriptase inhibitors – NRTI), while they provide long-term benefits by suppressing HBV viremia and reducing hepatic necroinflammation, they do not eliminate the cumulative risk of developing HCC (4–6). Therefore, while mortality caused by other widespread chronic infectious diseases – like tuberculosis or HIV – has declined over time, HBV-related mortality has increased by 22% in the last decade, highlighting the need to find new therapeutic strategies able to elicit a functional cure for chronic HBV infection.

A functional cure, defined as persistently undetectable levels of HBsAg in the absence of antiviral therapy (AVT) (7), implies the complete suppression of intrahepatic HBV replication even at the subclinical level. This level of viral control is observed during self-resolving acute HBV infection, suggesting that enhancement of HBV-specific immune responses through immunotherapy strategies could be a successful approach. Self-resolving HBV infection relies on an effective CD4 T cell, CD8 T cell and B cell response that will result in non-cytolytic HBV clearance. The critical antiviral role of cytotoxic CD8 T cells during this process has been demonstrated in the chimpanzee model (8) and it’s associated with vigorous, broad and polyclonal T cell responses (8–10). *In vitro* studies have shown that HBV-specific T cell-related production of IFNγ and TNF can effectively suppress viral replication (11). Critically, the immune system of liver transplant immune recipients is able to clear HBV infection (12, 13), proving that chronic HBV can be cured by a strong, broad and effective immune response.

In chronically infected patients a sufficient boost of HBV-specific immunity through immunotherapy can be challenging, due to both extremely low levels of HBV-specific T cells and weak T cell responses that are associated with immune exhaustion, immune dysregulation and inhibitory pathways of immune suppression [reviewed in (14)]. However, even with high exhaustion and low functionality there is an ongoing immune control during chronic HBV infection. This is highlighted by the fact that liver T cell infiltrates correlate with better viral control and less liver inflammation (15) and viral replication increases with immunosuppressive treatment (16). Thus, immunotherapies to boost both immune responses for chronic HBV infection hold promise and are being actively researched. It is worth noting that even if immunotherapy is now used in routine clinical practice and has even become the standard of care for some cancer indications [reviewed in (17)], the use of checkpoint inhibitors in the context of chronic viral infections is still controversial and in pre-clinical development stages [reviewed in (18)]. For HBV infection, most clinical data is in the context of HBV-induced HCC cancer treatment (19). Strong pre-clinical data clearly outlining whether the benefit of checkpoint blockade in chronic HBV-infected patients would outweigh the risk associated with this type of therapy is needed to encourage clinical trials aiming to a functional cure.

While immunotherapy strategies are intended to boost intrahepatic immunity, PBMCs are the most widely used proxy to study *in vitro* HBV-specific reactivity and efficacy of immunotherapies. In this approach, the scarcity of HBV-specific T cells within the PBMC compartment adds an additional challenge. To overcome this limitation, in our previous work (20) we developed a 5-day expansion protocol to increase sensitivity and we showed that PD-L1 blockade enhanced HBV-specific T cell reactivity. This approach, using expansion protocols to enrich on HBV-specific CD8 T cells prior to characterize their functionality, has been reported elsewhere (21, 22). Notwithstanding the relevance of this proof-of-concept, *in vitro* expansion and manipulation of the target cells can modify the expression of PD-1, PD-L1 and/or other activating (aR) or inhibitory (iR) receptors, affecting the *in vivo* translatability of the results. Thus, the aim of this study was to determine the efficacy of PD-L1 blockade *ex vivo* to increase the functionality of HBV-specific CD8 T cell responses.

## Results

### HBV-Specific CD8 T Cell Response Types Evolve With Clinical Progression

**Figure 1:**
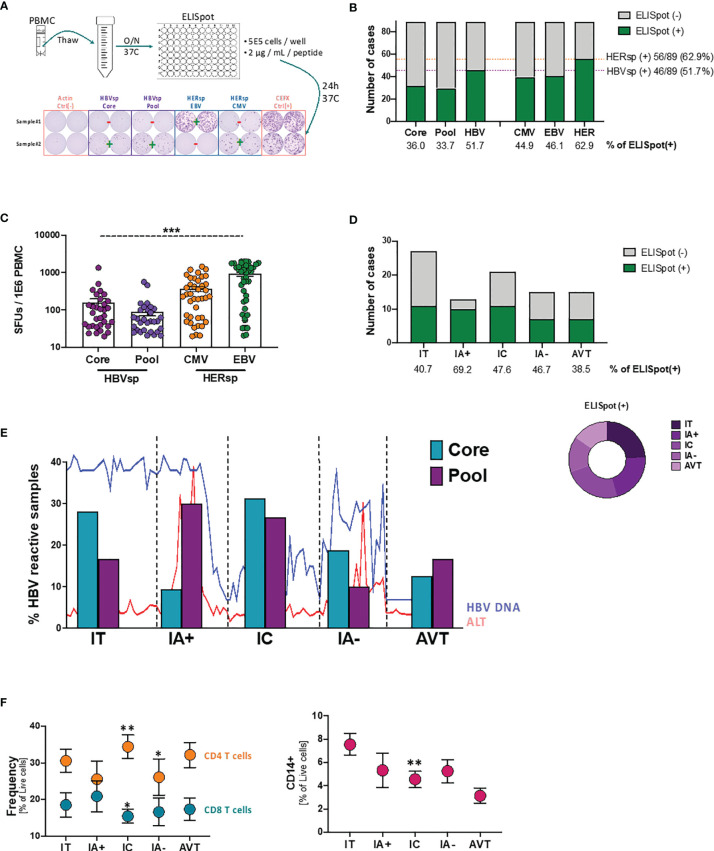
*Ex vivo* HBV-specific response evolves with clinical progression. **(A)** Schematic representation of the *ex vivo* ELISpot workflow with representative example of the response levels. **(B)** Number of samples with *ex vivo* ELISpot reactivity. HBV number of cases shows CORE and/or POOL while HER (HERPES) number of cases shows EBV and/or CMV. **(C)** Magnitude of the Ag-specific response, as measured as spot-forming units (SFUs) per million PBMCs, among the different stimulations. ***p < 0.0001; Kruskal-Wallis one-way analysis of variance. **(D)** Number of samples with *ex vivo* HBV-specific (core and/or pool) reactivity among the different clinical groups. **(E)** Evolution of the individual HBV-specific stimulations (CORE and POOL) with clinical progression. Lines show the variability of HBV DNA and ALT levels within each group. **(F)** Frequency of T cells (left panel) and CD14+ monocytes (right panel) among the different clinical groups. *p < 0.05; **p < 0.01; Mann Whitney U test. IT, Immune Tolerant; IA+, HBeAg+ Immune Active; IC, Immune Control; IA-, HBeAg- Immune Active; AVT, Anti-Viral Therapy.

To overcome the scarcity of HBV-specific CD8 T cells, in previous studies we (20), and others (21, 22), have used strategies focused on the expansion of HBV-specific T cells prior to PD-L1 blockade assessment. However, to avoid the modification of the expression patterns of the different inhibitory (iR) and activating (aR) receptors associated with expansion protocols, for this study we have optimized an *ex vivo* ELISpot strategy (**Figure 1A**). HBV-specific reactivity was analyzed using two different HBV peptide pools (HBVsp; Core and Pool). Reactivity to prevalent herpes infections (HERsp; CMV and EBV) was included for every sample as an example of chronic viral infections with effective immune control. Negative (Actin) and positive (CEFX) peptide pool controls were included for each sample. *Ex vivo* HBV-specific reactivity was detected in 36% and 33.7% of patients for Core and Pool, respectively (**Figure 1B**), with a combined HBV-specific response of 51.7% [HBVsp (+); 46/89; Core and/or Pool]. HERPES-specific reactivity [HERsp (+); 56/89; CMV and/or EBV] was detected at a slightly higher frequency (62.9%) but consistent with the prevalence of these infections on the general population in North America (23, 24). In addition, HBV-reactive samples showed a significantly lower magnitude of the response than HERPES-reactive samples (**Figure 1C**).

We then sought to analyze whether HBV-specific reactivity was associated with clinical parameters. Patients were categorized in 5 different groups according to HBeAg serology and ALT levels (25). According to this clinical definition Immune Tolerant (IT) is defined as HBeAg positive and ALT ≤ 1.3X upper limit normal (ULN); HBeAg+ Immune Active Hepatitis (IA+) as HBeAg positive and ALT > 1.3X ULN; *Immune control* (IC) as HBeAg negative and ALT ≤ 1.3X ULN and HBeAg- Immune Active hepatitis (IA-) as HBeAg negative and ALT > 1.3X ULN. All patients under antiviral therapy where included in the AVT group. *Ex vivo* HBV-specific reactivity was distributed among all clinical groups (**Figure 1D**) and independently of HBeAg, HBsAg, ALT or HBV DNA levels (**Supplementary Figure 1**). Interestingly, the proportion of the two different HBV stimuli changed with clinical evolution (**Figure 1E**). Our results show that during the IT phase, where the adaptive immune response is not strong, reactivity towards the more conserved proteins of the core and capsid were more frequently detected. HBV reactivity for patients with HBeAg+ hepatitis (IA+), a clinical status normally associated with a strong adaptive immune response that will lead to the negativization of the HBeAg, favored a broader and more polyclonal T cell response. This broad response is associated with the partial control of the HBV viral replication during the **IC** phase while the failure to maintain a diverse T cell response could be responsible of the viral escape observed during HBeAg- hepatitis (IA-). Patients under AVT, despite being a more heterogeneous group, consistently had a more constrained frequency similar to the reactivity observed during the IA- phase. In line with these results, peripheral CD8 T cells were increased in IA+ samples but significantly decreased in IC phase while inflammation [as measured as frequency of CD14+ monocytes (26)] consistently declines (**Figure 1F**). However, after viral escape, IA- samples fail to increase CD8 T cells or inflammation again. Altogether, these results strongly suggest that exhaustion of the HBV-specific CD8 T cell population plays an active role on the clinical progression of chronic HBV infection.

### General T Cell Exhaustion Is Not Detected in Chronic HBV Infection

**Figure 2:**
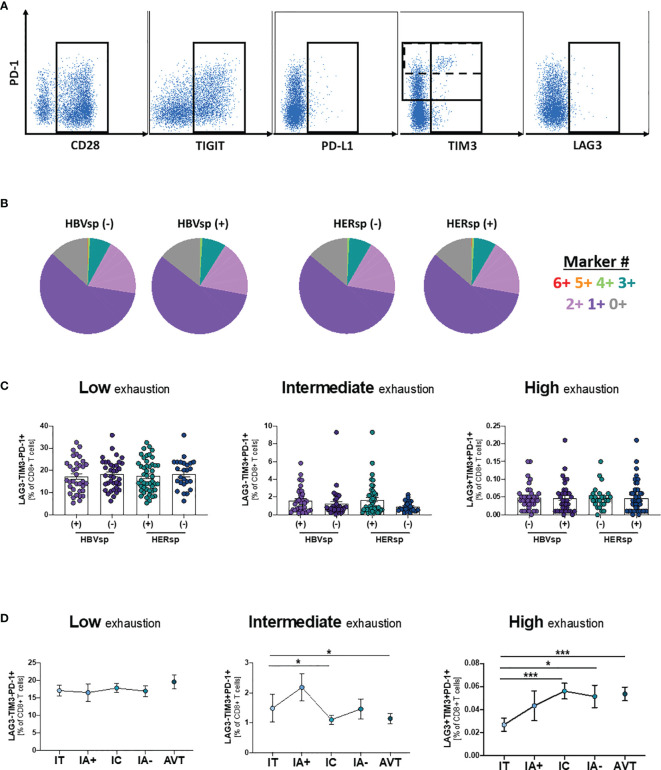
*Ex-vivo* Ag-specific reactivity does not associate with bulk CD8 T cell exhaustion levels. **(A)** Representative flow plots showing expression levels, on bulk CD8 T cells, of inhibitory (iR) and activating (aR) receptors included in the SPICE analysis. **(B)** SPICE analysis showing the distribution of marker co-expression (*6*+ markers for CD28+PD-1+TIGIT+PD-L1+TIM3+LAG3+) among patients with either HBV-specific reactivity (core and/or pool, left panel) or HERPES-specific reactivity (CMV and/or EBV, right panel). Frequency of CD8 T cells with phenotype consistent with low exhaustion (LAG3-TIM3-PD-1+, left panel), intermediate exhaustion (LAG3-TIM3+PD-1+, middle panel) or high exhaustion (LAG3+TIM3+PD-1+, right panel) **(C)** among patients with either HBV-specific reactivity (core and/or pool) or HERPES-specific reactivity (CMV and/or EBV) and **(D)** among the different clinical groups. IT, Immune Tolerant; IA+, HBeAg+ Immune Active; IC, Immune Control; IA-, HBeAg- Immune Active; AVT, Anti-Viral Therapy. *p < 0.05; ***p < 0.001.

To determine whether exhaustion had a main role in viral escape from CD8 T cell control, we quantified the expression of six different populations and 14 different iR and aR (full list of markers is shown in **Supplementary Table 2**). Representative examples of the gating strategy are shown in **Figure 2A** and **Supplementary Figure 2A**. Our data showed that neither the frequency of the different immune populations (CD11c DC, CD14 Monocytes, CD56 NK, CD20 B cells, CD4 T cells and CD8 T cells) nor iR and aR expression levels on bulk CD8 T cells was different between samples with or without *ex vivo* HBV-specific or HERPES-specific reactivity (data not shown). We then analyze expression patterns of six iR/aR strongly related with chronic HBV infection and PD-1/PD-L1 blockade immunotherapy (**Figure 2A**). Results showed that expression patterns of these selected markers were similar among samples with or without *ex vivo* HBV-specific or HERPES-specific reactivity (**Figure 2B**) and did not change with clinical progression (**Supplementary Figure 2B**). To confirm that exhaustion of bulk CD8 T cells was not associated with reactivity we quantified the frequency of CD8 T cells expressing a Low exhaustion (LAG3-TIM3-PD-1+), Intermediate exhaustion (LAG3-TIM3+PD-1+) or High exhaustion (LAG3+TIM3+PD-1+) phenotype, but no differences were observed between reactive or not reactive samples (**Figure 2C**). However, while the level of Low exhaustion remained unchanged among the different clinical groups (**Figure 2D**, left panel) we observed a significant decrease of CD8 T cells with Intermediate exhaustion phenotype (**Figure 2D**, middle panel) and a concomitant increase of Highly exhausted CD8 T cells (**Figure 2D**, right panel) associated with clinical progression. Any other immune population analyzed did not show changes associated with reactivity or clinical progression (data not shown), except for a significant increase of FAS-expressing CD11c DC during IA- phase (**Supplementary Figure 2C**). Increased sensitivity to cell death of antigen presenting cells like DC has been associated with failure to control chronic viral infections (27) and in this study was significantly associated with a lower frequency of peripheral CD8 T cells (**Supplementary Figure 2D**). This data suggests that even if exhaustion is related to longer times of infection and clinical progression and could be associated with the loss of HBV-specific reactivity leading to viral escape, antigen-specific CD8 T cells must be gathering most defects.

### HBV-Specific CD8 T Cells Express a Higher Frequency of Exhaustion Markers

**Figure 3:**
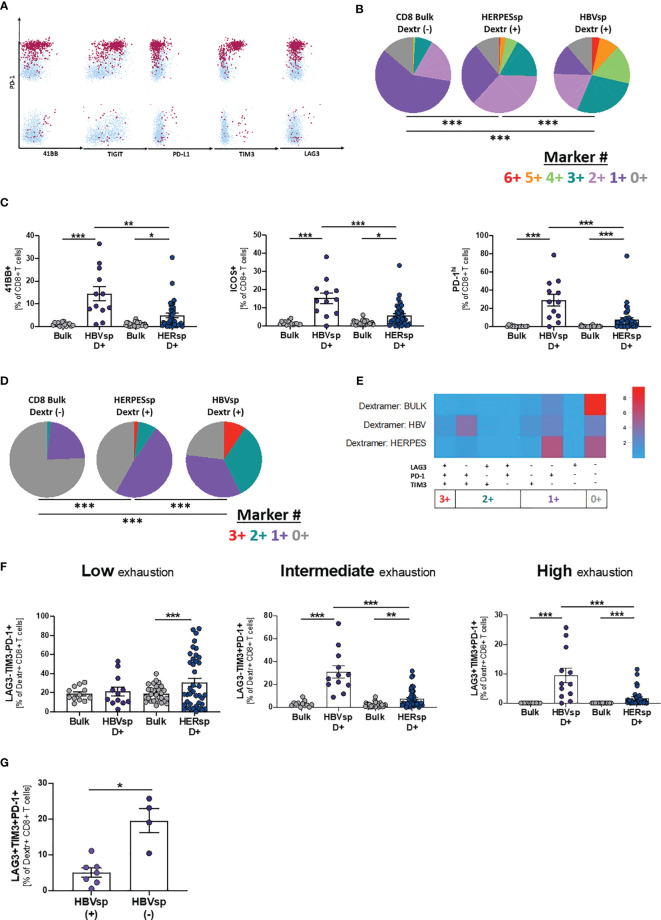
HBV-specific CD8 T cells express high frequency of exhaustion markers. **(A)** Representative flow plots showing expression levels, on HBV-specific CD8 T cells, of inhibitory (iR) and activating (aR) receptors included in the SPICE analysis. Dextramer-positive HBV-specific CD8 T cells (maroon) are overlaid on bulk CD8 T cells (light blue). **(B)** SPICE analysis showing the distribution of inhibitory receptors (*6*+ markers for 41BB+PD-1+TIGIT+PD-L1+TIM3+LAG3+) in bulk CD8 T cells (left pie), HERPES-specific CD8 T cells (CMV and/or EBV, *middle pie*) and HBV-specific CD8 T cells (core and/or pool, right pie). ***p < 0.0001; Permutation test. **(C)** Frequency of 41BB (*left panel*), ICOS (*middle panel*) and PD-1hi (right panel) expression in bulk, HBV-specific (core and/or pool) and HERPES-specific (CMV and/or EBV) CD8 T cells. *p < 0.05; **p < 0.001; ***p < 0.0001; Wilcoxon signed rank test (bulk *vs.* Ag-specific) and Mann-Whitney U (HBV-specific *vs.* HERPES-specific). Frequency of bulk, HBV-specific (core and/or pool) and HERPES-specific (CMV and/or EBV) CD8 T cells with phenotype consistent with low exhaustion (LAG3-TIM3-PD-1+, *1*+ markers), intermediate exhaustion (LAG3-TIM3+PD-1+, *2+ markers*) or high exhaustion (LAG3+TIM3+PD-1+, *3*+ markers) shown as **(D)** SPICE analysis; ***p < 0.0001; Permutation test, **(E)** SPICE CoolPlot and **(F)** pooled data; **p < 0.001; ***p < 0.0001; Wilcoxon signed rank test (bulk *vs.* Ag-specific) and Mann-Whitney U (HBV-specific *vs.* HERPES-specific). **(G)** Frequency of HBV-specific CD8 T cells with a high exhaustion phenotype (LAG3+TIM3+PD-1+) among patients with [HBVsp(+)] or without [HBVsp(-)] *ex vivo* HBV-specific reactivity (core and/or pool). *p < 0.05; Mann Whitney U test.

Dextramer positivity was tested for any sample with the alleles HLA-A*0201; B*3501 and B*5101 (n = 48). MHC I Dextramers^®^ (Immundex) specific for HBV capsid, three different epitopes of HBV Protein S, CMV and EBV were used to detect Ag specific T cells. A gating example for Ag-specific CD8 T cells is shown in **Supplementary Figure 3A**. Fourteen individual patients had detectable HBV-specific CD8 T cells using this method. The positive samples were distributed equally among clinical groups (**Supplementary Figure 3B**) and independent of HBsAg levels (**Supplementary Figure 3C**). HBV DNA levels were slightly increased in the samples with detectable HBV-specific CD8 T cells (**Supplementary Figure 3C**). We then quantified the expression of iR and aR in dextramer-positive Ag-specific CD8 T cells (**Figure 3A** and **Supplementary Figure 3A**). Expression patterns of 6 different receptors (**Figure 3B**, 6+ markers PD-1+41BB+TIGIT+PD-L1+TIM3+LAG3+) showed that Ag-specific CD8 T cells, either HERPES-specific or HBV-specific, are significantly more exhausted than paired bulk CD8 T cells. In addition, HERPES-specific CD8 T cells, which are successfully controlling viral replication, are significantly less exhausted than the HBV-specific CD8 T cells (**Figure 3B**). While HERPES-specific CD8 T cells express mostly one or two of these iR at the same time (**Figure 3B**, 2+/1+ makers), most HBV-specific CD8 T cells simultaneously co-express 3 to 6 iR. In addition, the frequency of some receptors (**Supplementary Figure 3D**; CD127, 2B4, TIGIT, PD-L1 and PD-L2) is significantly different between bulk CD8 and Ag-specific CD8 T cells but similar between HERPES- and HBV-specific CD8 T cells. However, HBV-specific CD8 T cells showed significantly higher expression of markers like 4-1BB, ICOS or PD-1^hi^ (**Figure 3C**).

When we focused on PD-1, TIM3 and LAG3 expression, hallmarks of functional exhaustion, the data showed a significant increase of Low exhaustion and Intermediate exhaustion HERPES-specific CD8 T cells (1+ markers; LAG3-TIM3-PD-1+ and 2+ markers; LAG3-TIM3+PD-1+), phenotypes consistent with an activated and functional response associated to viral control (**Figures 3D–F**). HBV-specific CD8 T cells, on the other hand, showed a significant increase of Intermediate exhaustion and High exhaustion (2+ markers; LAG3-TIM3+PD-1+ and 3+ markers; LAG3+TIM3+PD-1+), a phenotype consistent with functional exhaustion and lack of viral control (**Figures 3D–F)**. Despite the low number of samples, High exhaustion tends to increase with clinical progression (**Supplementary Figure 3E**). In line with these results, patients with *ex vivo* HBV reactivity [HBVsp(+)] have significantly lower amounts of highly exhausted HBV-specific CD8 T cells (**Figure 3G**).

**Figure 4:**
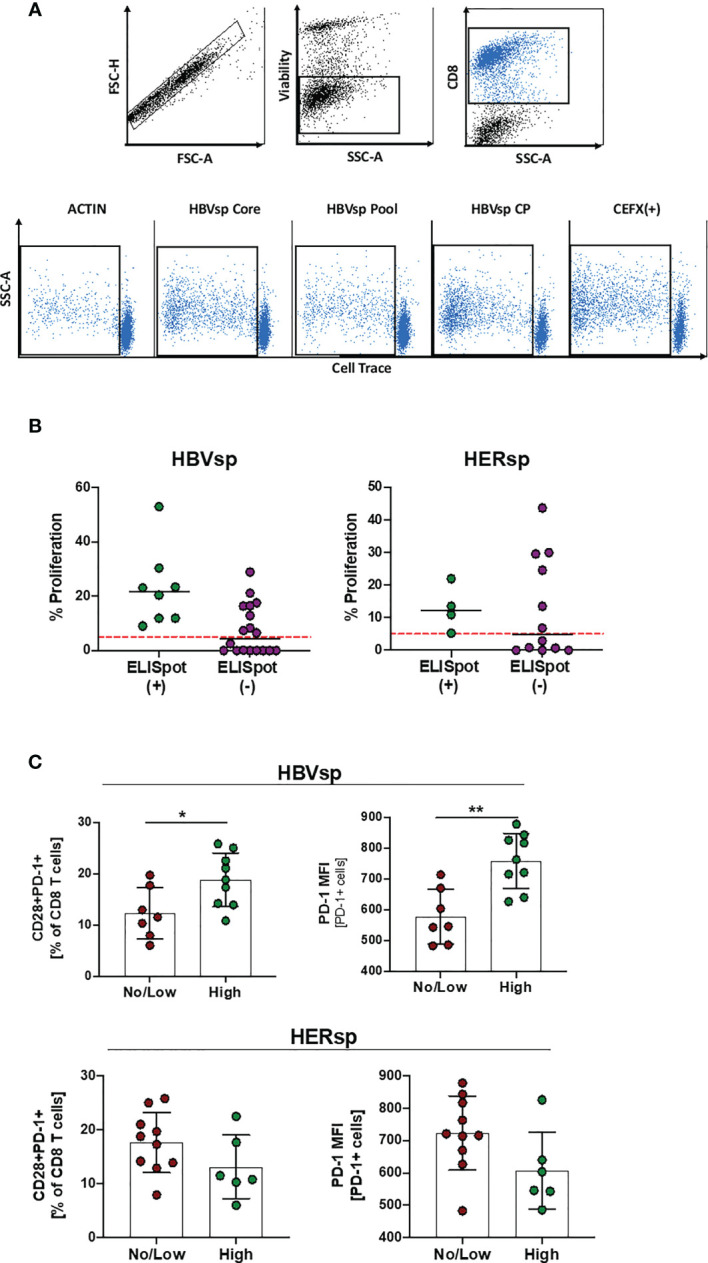
T cell expansion unveils Ag-specific T cells in *ex vivo* not-reactive samples. **(A)** Representative flow plots showing the gating strategy for the cell trace dilution assay. **(B)** Frequency of CD8 proliferating cells among reactive and non-reactive samples for both HBV-specific (core and/or pool) and HERPES-specific (CMV and/or EBV) assays. **(C)** Baseline frequency of CD28+PD-1+ CD8 T cells and the mean fluorescence intensity (MFI) of the PD-1 marker on CD8 T cells among samples with low or high proliferation levels. *p < 0.05; **p < 0.001; Mann Whitney U.

We then sought to determine whether non-reactive, exhausted HBV-specific CD8 T cells retained the ability to proliferate. Samples with different levels of *ex vivo* HBV reactivity were incubated, in the presence of IL-2, with different HBV peptide pools and controls and proliferation levels were quantified on day 10 (**Figure 4A**). Samples with *ex vivo* reactivity [ELISpot(+)] showed consistent proliferation for both HBV- and HERPES-specific stimulations (**Figure 4B**). Samples that did not show any *ex vivo* reactivity for the ELISpot assay were only partially recovered by proliferation (**Figure 4B**). Despite the lower limit of detection of the proliferation assay, some samples did not show any proliferative capability. High proliferation levels for HBV-specific T cell reactivity was associated with a higher baseline frequency of CD28+PD-1+ co-expression and higher expression of PD-1 per cell on bulk CD8 T cells (**Figure 4C** and **Supplementary Figure 4A**), suggesting that low exhaustion is a requisite for HBV-specific T cell proliferation, while this difference was not observed for HERPES-associated proliferation. Altogether, these results show that functional exhaustion of HBV-specific CD8 T cells plays a role in the clinical progression of chronic HBV infection and could be a barrier to successful immunotherapy strategies.

### Lack of Response to PD-L1 Blockade Is Associated With Higher Frequency of Functionally Exhausted HBV-Specific T Cells

**Figure 5:**
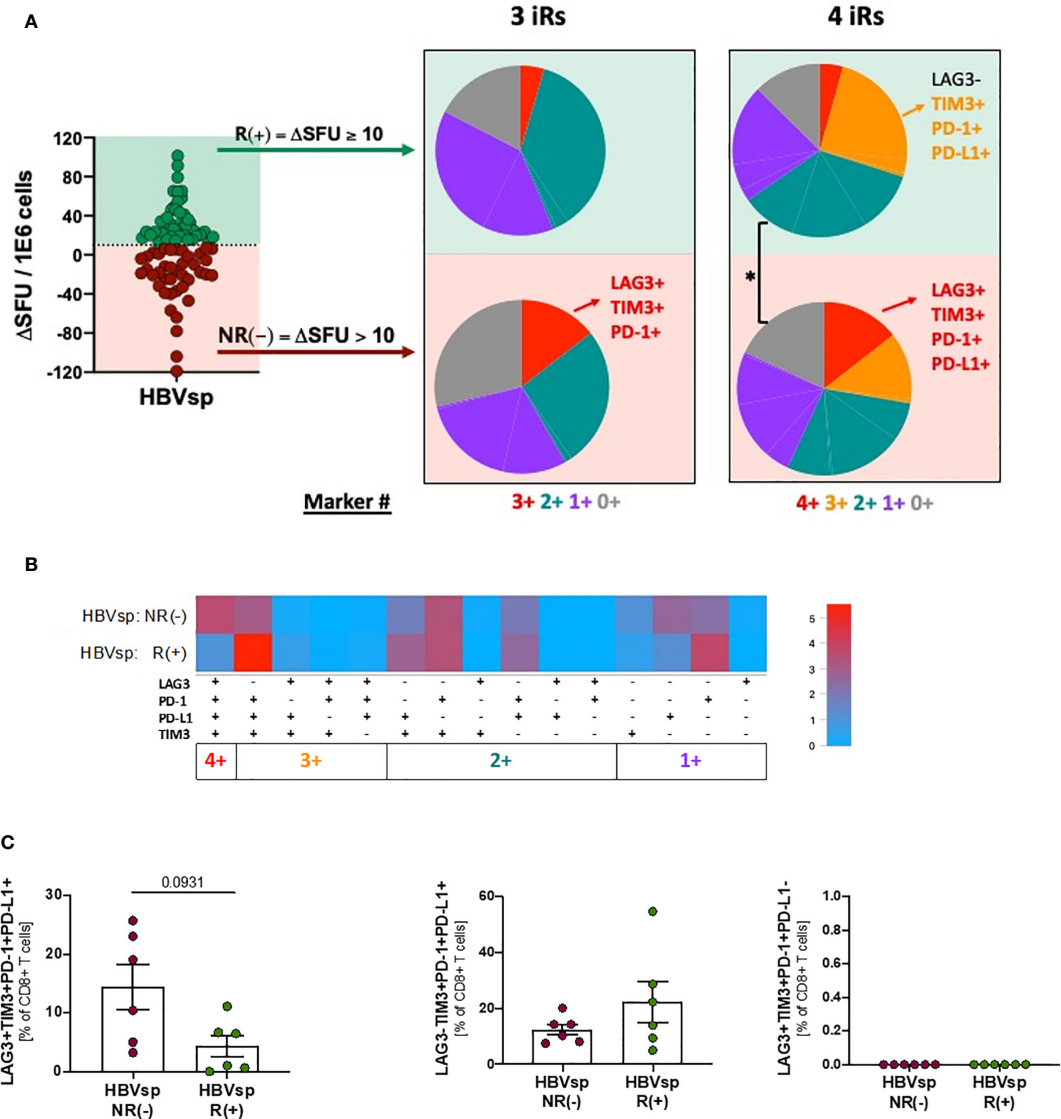
Highly exhausted HBV-specific CD8 T cells do not respond to PD-L1 blockade. Frequency of HBV-specific (core and/or pool) CD8 T cells with phenotype consistent with low exhaustion (LAG3-TIM3-PD-1+PD-L1-; *1*+ markers); intermediate exhaustion LAG3-TIM3+PD-1+PD-L1±; *2+/3+* markers) and high exhaustion (LAG3+TIM3+PD-1+PD-L1+; *4*+ markers) between responders (R) and non-responders (NR) to PD-L1 blockade shown as **(A)** SPICE analysis. *p < 0.05; Permutation test, **(B)** SPICE CoolPlot and **(C)** pooled data. Mann Whitney U.

*Ex vivo* HBV-specific reactivity was assessed in the presence of MEDI2790, a PD-L1 inhibitor. Response to PD-L1 blockade was defined as increase of at least 10 SFUs in the *ex vivo* ELISpot when compared to the untreated control. We observed a 50% response to MEDI2790 [R(+)](**Figure 5A**). Response to PD-L1 blockade was independent of HBeAg, HBsAg, ALT or HBV DNA levels (**Supplementary Figures 5A, B**). Distribution patterns of the 6 iR (**Supplementary Figure 5C**, 6+ markers PD-1+41BB+TIGIT+PD-L1+TIM3+LAG3+) was also similar between responders and not responders. However, non-responders had a significantly higher frequency of functionally exhausted HBV-specific CD8 T cells (3+ markers, LAG3+TIM3+PD-1+), independently of the expression levels of PD-L1 (**Figures 5A–C**) while response to MEDI2790 was associated with a higher frequency of the phenotype TIM-3+PD-1+PD-L1+ in absence of LAG3 expression (**Figures 5A, C**). These results strongly suggest that LAG3 expression signifies a functionally exhausted status that cannot be recovered by PD-L1 blockade strategies.

### Response to PD-L1 Blockade Increases HBV-Specific T Cell Functionality

**Figure 6:**
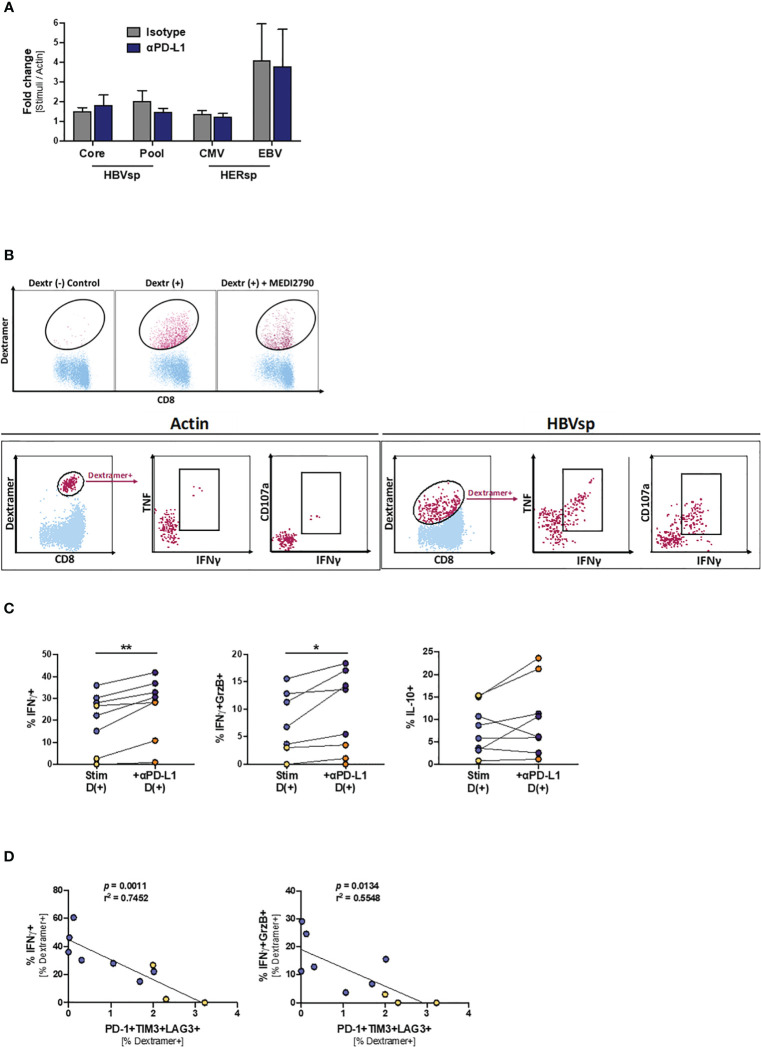
PD-L1 blockade increases the frequency of IFNγ-producing Ag-specific CD8 T cells. **(A)** Effect of PD-L1 blockade on the proliferation levels of Ag-specific CD8 T cells. **(B)** Representative flow plots showing the gating strategy for intracellular cytokine staining of dasatinib-treated HBV-specific CD8 T cells. Dextramer-positive CD8 T cells (maroon) are overlaid on bulk CD8 T cells (light blue). While cytokines cannot be detected on samples stimulated with Actin negative control (left panel), dasatinib-treated stimulated HBV-specific T cells accumulate both cytokines and degranulation markers (right panel). **(C)** Effect of PD-L1 blockade in IFNγ (left panel), IFNγ+GrzB+ (middle panel) and IL-10 (right panel) production. Blue dots show HERPES-specific stimulations (CMV or EBV) while orange dots show HBV-specific stimulations (pool). *p < 0.05; **p < 0.001; Wilcoxon signed rank test. **(D)** Linear regression showing the negative correlation between Ag-specific IFNγ production and the frequency of highly exhausted (LAG3+TIM3+PD-1+) Ag-specific CD8 T cells. Blue dots show HERPES-specific stimulations (CMV or EBV) while orange dots show HBV-specific stimulations (pool).

Finally, we tried to determine the mechanisms underlying the increase of *ex vivo* HBV reactivity after PD-L1 blockade. However, proliferation levels were not increased in the presence of MEDI2790 (**Figure 6A**), MitoTempo, a mitochondrial-targeted antioxidant agent that has shown to restore T cell activation (28)IL-12, a third signal cytokine that regulates T cell responses and could rescue anti-viral activity of exhausted cells (29, 30), or the combination of all three agents (**Supplementary Figure 6A**). Then, we sought to determine whether PD-L1 blockade would affect cytokine production. HBV-specific CD8 T cells are present in such a low frequency that conventional approaches combining peptide pool stimulation and intracellular staining of bulk CD8 T cells often fail because the signal is under the threshold of detection. To overcome this limitation, we used an innovative approach to force the upregulation of the TCR to the cell surface that allowed us to combine dextramer staining, peptide pool stimulation and intracellular staining of HBV-specific CD8 T cells, greatly increasing the sensitivity of the flow cytometry-based approach. As shown in **Figure 6B**, overnight stimulation of PBMCs with the irrelevant peptide Actin and dasatinib treatment prior to the dextramer and intracellular staining did not detect cytokine production among HBV-specific CD8 T cells. However, when a relevant peptide (HBV Pool) was used for the stimulation (**Figure 6B**, right panel) we were able to detect a robust cytokine production by HBV-specific CD8 T cells. PD-L1 blockade significantly increased IFNγ production and cytotoxicity (IFNγ+GrzB+) for both HBV-specific and HERPES-specific CD8 T cells (**Figure 6C**, left and middle panels). IL-10 production was increased only for HBV-specific T cells (**Figure 6C**, right panel) while TNF, IL-2 and IFNγ+CD107a+ remained unchanged (**Supplementary Figure 6B**). In line with our previous results, the frequency of Ag-specific CD8 T cells with a functional response (cytokine production or cytotoxicity) is inversely associated with the frequency of functional exhaustion (**Figure 6D** and **Supplementary Figure 6C**).

## Discussion

In this study we show that a high frequency of functionally exhausted HBV-specific CD8 T cells (as determined by the simultaneous expression of LAG3+TIM3+PD-1+) is associated with both lack of *ex vivo* reactivity and unresponsiveness to PD-L1 blockade. These results could have implications in the design of immunotherapy strategies aiming to achieve a functional cure for chronic HBV infection.

Chronic HBV infection, besides the stigma and significant impact on quality of life (31, 32), is characterized by a 100-fold increase of the risk to develop hepatocellular carcinoma (HCC) (2, 33). A functional cure, defined as persistent absence of HBsAg in the absence of antiviral therapy, indicates complete immune control and viral suppression and is regarded as the optimal point of therapy (7). Current antiviral therapy (AVT) with different nucleos(t)ide reverse transcriptase inhibitors (NRTI) can provide long-term benefits by suppressing HBV viremia but they are still life-long treatments with negligible rates of HBsAg loss and patients maintain a 6% 8-years cumulative risk to develop HCC (4–6). Some patients can achieve functional cure after discontinuation of NRTI treatment (34, 35), but this is a minority and stopping NRTI’s is not without risks as severe flares and liver decompensation may occur (36). PEGylated-IFNα is a finite but toxic therapy, given for 6-12 months as monotherapy or combined with NRTI, resulting in about 10% HBsAg loss after long-term follow-up. Thus, achieving HBsAg loss with finite therapy is a current need for chronic HBV infected patients.

The immune system is able to completely suppress intrahepatic HBV replication during self-resolving acute HBV infection and the critical antiviral role of adaptive immunity, especially cytotoxic CD8 T cells, during the resolution of acute HBV infection has been clearly defined (8–10). In humans, liver transplant immune recipients are able to clear HBV infection in organs from chronically infected donors (12, 13), proving that chronic HBV infection can be functionally cured by an effective immune response. Direct disruption of the PD-1:PD-L1 axis with monoclonal antibodies has been successfully tested to restore functionality of CD8 T cell in both, cancer and infectious diseases (37–40). The combination of immunotherapy and therapeutic vaccine showed some promising results in a small clinical trial (41). In addition, we have previously shown that *in vitro* PD-L1 blockade with MEDI2790 increased by two-fold the HBV-specific T cell response in 97% of chronically infected patients with baseline T cell reactivity (20). However, due to the extremely low frequency of peripheral HBV-specific T cells, in our past study HBV-specific T cell were expanded prior to the PD-L1 blockade, serving as a proof-of-concept for the use of immunotherapy in chronic HBV infection.

To increase the translatability of this proof-of-concept, in this study we optimized an *ex vivo* approach to determine HBV-specific T cell reactivity and response to MEDI2790. Two different HBV peptide pools, a commercially available peptide pool containing 9 HLA-class-I restricted T cell epitopes known to be highly immunogenic and a custom-generated pool including 34 HLA-class-I restricted epitopes shared by all HBV genotypes, were used to maximize the level of response when using this *ex vivo* approach. HBsAg overlapping peptide pools were not included for *ex vivo* ELISpot analysis, since HBsAg immune responses are normally scarce and impaired during chronic HBV (22, 40, 42). As recently reported (43, 44), in this study we show that the immune response leading to the initial control of viral replication is broad and targets multiple HBV proteins while HBeAg- Immune Active hepatitis is characterized by a narrow and weak HBV-specific T cell response. In line with these results, we found that HBV-specific CD8 T cells are significantly more exhausted than herpes-specific CD8 T cells (CMV and/or EBV) in the same patients. It is worth noting that herpes infections are also chronically established but, in immunocompetent hosts, don’t show a clinical progression due to complete control of replication by the immune system. Not surprisingly, high levels of functional exhaustion in the HBV-specific CD8 T cell population was associated to lack of *ex vivo* reactivity and failure to respond to MEDI2790 PD-L1 blockade.

We then developed a strategy to quantify cytokine production by HBV-specific CD8 T cell by flow cytometry. While MHCI dextramers have been widely used as a detection tool for *ex vivo* approaches, once antigen-specific CD8 T cells are activated the T cell receptor gets internalized and thus unavailable for dextramer-based staining. Due to this limitation, flow cytometry approaches to quantify cytokine production from antigen-specific T cells usually focus on the bulk CD8 subset, greatly limiting the sensitivity of the detection. This is especially problematic in chronic HBV infection where, due to the rare frequency of these cells, the frequency of cytokine-producing cells is rarely detected over the background noise. To overcome this limitation, we used dasatinib, a blocker of the Src kinase family (45–47), to force the re-expression of the TCR on the surface of antigen-specific CD8 T cells after peptide stimulation. Using this approach, we were able to determine that HBV-specific CD8 T cells with low level of exhaustion were indeed responding to PD-L1 blockade by increasing the production of IFNγ and IFNγ+GrzB+ cytotoxic responses. However, HBV-specific CD8 T cells with higher levels of exhaustion, mainly characterized by the expression of LAG3, are unable to respond to this immunotherapeutic approach.

Bengsch et al. (48) have previously shown that iR expression on HBV-specific CD8 T cells is hierarchical, with PD-1 being expressed the most and LAG-3 being the hardest iR to detect. Despite the comprehensive characterization of the different iR expression on HBV-specific CD8 T cells, they analyzed the response to PD-L1 blockade as the increase on proliferative capacity. Since this approach has a higher detection limit, they were not able to identify a specific phenotype associated with lack of response. In this study, we were able to determine that the co-expression of PD-1, TIM-3 and LAG-3 is associated with the lack of response to anti-PD-L1 antibodies. This result has direct translational implications. Foremost, it suggests that monotherapy strategies are not suitable to treat chronically infected HBV patients. In addition, since terminally exhausted CD8 T cells undergo epigenetic changes that prevents for this phenotype to be rescued (49, 50), finding the appropriate therapeutic window before HBV-specific T cells become functionally exhausted, may be necessary to successfully apply immunotherapy to cure chronic HBV infection. Finally, even if combination strategies may be still effective in selected or unselected HBV patients, targeting LAG-3 blockade (51, 52) may be essential to elicit a clinically effective response.

Altogether, our results suggest that while immunotherapeutic approaches aimed to boost HBV-specific T cell immunity could be a successful strategy to elicit a functional cure for chronic HBV infection, a monotherapy aiming to disrupt the PD-1:PD-L1 axis could have limited effectiveness. The high levels of functional exhaustion present in the HBV-specific CD8 T cell subset suggest that combination strategies, potentially including the combination of anti-LAG-3 with other anti-iR antibodies, should be explored and likely prioritized for unselected chronic HBV patients.

## Methods

### Patients

One hundred adult patients with chronic HBV infection (female 38%; median age 48 years old) were included in this study. All patients were in follow up at the Toronto General Hospital Liver Centre, University Health Network in Toronto, Canada and willing to provide informed consent. All patients had confirmed chronic HBV infection, documented by the presence of HBsAg for at least 12 months prior to the inclusion of this study. The study protocol was approved by the Ethics Committee of the Toronto General Hospital, University Health Network. Exclusion criteria included acute HBV infection, acute flare or reactivation of HBV infection (defined as symptoms of acute hepatitis and recent elevation of ALT > 10xULN or bilirubin levels), treatment with interferon (IFN)-α, systemic corticosteroids or any other immune modulators or suppressive agents within 4 weeks of screening, cirrhosis, hepatocellular carcinoma, liver transplantation, known coinfection with HCV, HDV and/or HIV, need for renal dialysis and known active autoimmune disease, including autoimmune hepatitis. Informed consent was obtained from each individual at enrollment. Peripheral blood samples were obtained by venipuncture, anonymized and processed to obtain peripheral blood mononuclear cells (PBMC). All samples were cryopreserved until further use. Characteristics of the cohort are summarized in **Table 1**.

**Table 1:** Characteristics of the cohort.

	Flow cytometry	*Ex vivo* ELISpot	*p*
n	100	89	
Sex (% Female)	38/100 (38%)	44/89 (49.4%)	
Age	48 [35 - 57]	44 [33 – 54]	NS^1^
Race (% Asian)	81/100 (81%)	76/89 (85.4%)	
AVT^2^	21/100 (21%)	13/89 (14.6%)	
AVT (years)	5 [3.5 – 7.8]	5 [3.5 – 8.7]	NS
HBeAg (% Negative)			
AVT(-)	49/79 (62.0%)	36/76 (47.4%)	
AVT(+)	18/21 (85.7%)	10/13 (76.9%)	
ALT (U/mL)	30 [23 - 58]	29 [22 – 63]	NS
ULN^3^ ≤ 1.3X			
AVT(-)	54/79 (68.4%)	49/76 (64.5%)	
AVT(+)	18/21 (85.7%)	13/13 (100%)	
Log HBV DNA	4.0 [1.3 – 8.0]	5.8 [2.3 – 8.2]	NS
HBsAg (IU/mL)	2803 [683 - 12176]	5181 [1023 – 45385]	NS
HBsAg (% ≤10)			
AVT(-)	6/68 (8.8%)	4/64 (6.3%)	
AVT(+)	1/20 (5.0%)	0/13 (0.0%)	
Clinical groups^4^			
* Immune Tolerant* (IT)	19/100 (19%)	27/89 (30.3%)	
* HBeAg+ Immune Active* (IA+)	12/100 (12%)	13/89 (14.6%)	
* Immune Control* (IC)	34/100 (34%)	21/89 (23.6%)	
* HBeAg- Immune Active* (IA-)	14/100 (14%)	15/89 (16.9%)	
* Antiviral Therapy* (AVT)	21/100 (21%)	13/89 (14.6%)	

^1^NS, Not significant; p > 0.1. ^2^AVT, Antiviral Therapy. ^3^ULN, ALT upper limit of normal. ^4^All HBeAg-negative patients in this category spontaneously seroconvert. IT = HBeAg (+) and ULN ≤ 1.3X; HBeAg+ IA = HBeAg (+) and ULN > 1.3X; IC = HBeAg (-) and ULN ≤ 1.3X; HBeAg- IA = HBeAg (-) and ULN > 1.3X. Dichotomic variables are expressed as number/total number (frequency). Continuous variables are expressed as median [Interquartile range, IQR].

### *Ex Vivo* ELISpot

PBMC samples from chronic hepatitis B (HBV) patients were thawed and washed in CTL Anti-Aggregate™ medium (Cellular Technology Limited, CTL) and rested overnight at 37°C in complete RPMI with 10% human serum. Duplicated wells containing 5E5 PBMC were incubated overnight with the appropriate peptide pools in CTL-Test™ Medium (Cellular Technology Limited, CTL), including negative and positive controls, in the presence of MEDI2790 or a control IgG isotype. Antigen-specific T cell responses were quantified using ELISpot PLUS IFNγ pre-coated plates (MabTech) and the ImmunoSpot^®^ reader and software (Cellular Technology Limited, CTL). CORE, POOL, CMV, EBV and the negative control ACTIN pool were used at a concentration of 2 µg/peptide/mL. The positive control CEFX pool was used at 0.2 µg/peptide/mL. HBVsp refers to combined results from HBV CORE and HBV POOL stimulations. HERsp (HERPES) refers to combined results from CMV and EBV stimulations. Additional information, including specific peptides, of the peptide pools can be found in **Supplementary Table 1**.

One hundred samples were tested in this study. Samples with failed positive and/or negative controls after two independent attempts were discarded. A total of 89 ELISpot quantifications passed all quality controls and were included in the analysis for this study. All results are reported after background subtraction using each sample’s actin control.

### Polychromatic Flow Cytometry

#### *Ex Vivo* Analysis of Bulk CD8 T Cells

PBMC samples from the 100 chronic HBV patients were thawed, washed in CTL Anti-Aggregate™ medium (Cellular Technology Limited, CTL) and rested 2h in CTL Test™ medium (Cellular Technology Limited, CTL). Cells were then washed and stained in 96-well plates at 1E6 - 2E6 cells per well with titrated amounts of LIVE/DEAD™ Fixable Blue Dead Cell stain kit (Invitrogen). All surface markers were stained at a final volume of 100µL with titrated amounts of monoclonal antibodies, in the presence of Super Bright Staining Buffer (eBiosciences) for 45 min at room temperature. After the final wash cells were fixed with 1% PFA/PBS and acquired in a BDSymphony^®^ flow cytometer. Analysis was performed with FlowJo v10.6.2. Further information about the antibodies can be found in **Supplementary Table 2**.

#### *Ex Vivo* Analysis of HBV-Specific CD8 T Cells

For a subset of patients with selected HLA alleles (HLA-A*0201, HLA-B*3501 and HLA-B*5101; n = 48) dextramer staining was included in the *ex vivo* analysis. Samples were incubated for 15 min, previous to the surface marker staining, with the appropriated dextramers depending on their HLA type. Negative controls were included for all samples. Further information of the dextramers can be found in **Supplementary Table 3**.

#### Functional Analysis by Intracellular Staining

Thirteen HLA-A*0201 samples, with HBV-specific dextramer frequency higher than 0.1% and sample availability were selected for a functional analysis. PBMC samples were thawed, washed in CTL Anti-Aggregate™ medium (Cellular Technology Limited, CTL) and rested 2h in CTL Test Plus™ medium (Cellular Technology Limited, CTL). PBMC were then washed and added to 48 well plates at a concentration of 2E6/mL on CTL Test Plus™ medium. Samples were stimulated with either HBV Pool (PX-HBV ThinkPeptide), CMV (PA-CMV-001, PanaTecs), EBV (PA-CMV-001, PanaTecs) or Actin control (PM-ACTS, JPT) at 2µg/peptide/mL for 12h in the presence of brefeldin A (BFA) and titrated amounts of CD107a antibody. Cells were then washed and incubated with Dasatinib 100mM (Sigma-Aldrich) in PBS for 1h at 37°C to favor the re-expression of the TCR in the cell surface (45–47). All further incubations and washed were done in the presence of 100mM Dasatinib (Sigma-Aldrich). This approach is a modification of a previously reported protocol (30, 53). After the incubation cells were stained with titrated amounts of LIVE/DEAD™ Fixable Blue Dead Cell stain kit (Invitrogen). After a blocking step, each actin control/peptide-stimulated pair was stained with the appropriate dextramers (Capsid or Protein S for Actin/HBV pool, CMV for Actin/CMV pool and EBV for Actin/EBV pool) for 15 minutes. Stimulated and dasatinib-treated samples stained with control dextramers were used to determine the gating strategy. All surface markers were then stained at a final volume of 100µL with titrated amounts of monoclonal antibodies in the presence of Super Bright Staining Buffer (eBiosciences). Cells were then permeabilized using a fixation/permeabilization kit (BD) according to the manufacturer’s instructions and stained with titrated amounts of monoclonal antibodies for 45 min at 4°C. After the final washes cell were resuspended in 1% PFA/PBS and acquired in a BDSymphony^®^ flow cytometer. Analysis was performed with FlowJo v10.6.2. Further information about the antibodies and dextramers can be found in **Supplementary Tables 2, 3**.

### Proliferation Assay

Fifteen samples with available Leukopak where included for the proliferation assay. PBMCs were thawed, washed in CTL Anti-Aggregate™ medium (Cellular Technology Limited, CTL) and rested for 2h at 37°C in CTL Test Plus™ medium (Cellular Technology Limited, CTL). After resting PBMCs were counted and transferred to 50mL conical tubes and resuspended at 1E6/mL in PBS with titrated amounts of Cell Trace™ Violet Cell proliferation (ThermoFisher Scientific). After a 20min incubation at 37°C the staining was stopped with PBS 10% FCS at 1:4 ratio. PBMCs stained with the Violet Cell Trace™ were cultured at 2E6/mL, in duplicate, in the presence of 20 IU of IL-2 and 2µg/peptide/mL of the appropriate stimuli (HBV Core, HBV Pool, HBV Capsid, CMV, EBV, Actin or CEFX peptide pools as described in the ELISpot section) in the presence of either 1) IgG control, 2) MEDI2790, 3) 0.1 μM Mitotempo (Sigma Aldrich), 4) 10 ng/mL IL-12 and 5) MEDI2790 + 0.1 μM Mitotempo (Sigma Aldrich) + 10 ng/mL IL-12. Media (with IL-2) was changed at day 5 and proliferation was assessed on day 10 by flow cytometry.

### MEDI2790 Antibody Generation

MEDI2790 generation has been previously described (20, 54). Briefly, IgG2 and IgG4 XenoMouse animals were immunized with human PD-L1-Ig or CHO cells expressing human PD-L1. Hybridomas were established and supernatants screened for binding to human PD-L1-transfected HEX 293 cells and inhibition of PD-1 binding to PD-L1 expressing CHO cells. MEDI2790 was selected based on affinity, activity and specificity profile. The constant domain of the antibody was then exchanged for a human IgG1 triple-mutant domain containing three-point mutations that reduce binding to C1q and Fc gamma receptors, resulting in reduced antibody-dependent cellular cytotoxicity (ADCC) and complement-dependent cytotoxicity (CDC).

### Statistical Analysis

GraphPad Prism (PRISM 8 for macOS v8.3.0) was used to perform statistical analyses and to create graphs. Data is shown as number of cases, individual points or bars depicting the mean ± standard error (SEM). Variables were analyzed using non-parametric tests as appropriate: Mann-Whitney U test for unpaired variables, Wilcoxon matched-pairs signed rank test for paired variables and Kruskal-Wallis one-way analysis of variance for the simultaneous comparison of 3 or more groups. Associations were analyzed by linear regression and 95% confident interval (CI). Analysis and graphical representation of the distribution of inhibitory (iR) and activating (aR) receptors on the different CD8 T cell populations was performed using the Simplified Presentation of Incredibly Complex Evaluations (SPICE v6) as previously described (55).

## Data Availability Statement

The original contributions presented in the study are included in the article/**Supplementary Material**. Further inquiries can be directed to the corresponding author.

## Author Contributions

SFM and SR designed the study. AG, JF and HJ provided and processed the samples. SFM, AS and EL performed the experiments. SFM and AS performed the analysis. SFM and SR wrote the manuscript. All authors contributed to the article and approved the submitted version.

## Funding

The study was fully funded by AstraZeneca.

## Conflict of Interest

Authors SFM, ASB, EL and SHR were employed by company AstraZeneca, involved in the development and production of MEDI2790.

The authors declare that this study was funded by AstraZeneca. The funder had the following involvement in the study: development and production of MEDI2790.

## Publisher’s Note

All claims expressed in this article are solely those of the authors and do not necessarily represent those of their affiliated organizations, or those of the publisher, the editors and the reviewers. Any product that may be evaluated in this article, or claim that may be made by its manufacturer, is not guaranteed or endorsed by the publisher.
