# Supplementary material for: Functional Exhaustion of HBV-Specific CD8 T Cells Impedes PD-L1 Blockade Efficacy in Chronic HBV Infection
Source: Front Immunol. 2021 Sep 13;12:648420. doi: 10.3389/fimmu.2021.648420 (PMC8473828; doi:10.3389/fimmu.2021.648420)
Supplement: Supplementary file 1 [file DataSheet_1.docx]

**Functional exhaustion of HBV-specific CD8 T cells impedes anti-PD-L1 blockade efficacy in chronic HBV infection**

Sara Ferrando-Martinez et al.

**Supplementary material**


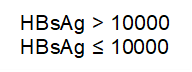


**Supplementary Figure 1. A)** Number of samples with *ex vivo* HBV-specific reactivity (core and/or pool) according to HBeAg seroconversion status (*left panel*), HBsAg levels (*middle panel*) and ALT levels (*right panel*). **B)** Pooled data showing HBV DNA (*left panel*), HBsAg (*middle panel*) and ALT (*right panel*) levels among patients with [HBVsp(+)] or without [HBVsp(-)] *ex vivo* HBV-specific reactivity (core and/or pool).

**Supplementary Figure 2.** **A)** Representative flow plots showing expression levels, on bulk CD8T cells, of other inhibitory (iR) and activating (aR) receptors included in the flow cytometry analysis. **B)** SPICE analysis showing the distribution of marker co-expression (*6+ markers* for CD28+PD-1+TIGIT+PD-L1+TIM3+LAG3+; *0+ markers* for CD28-PD-1-TIGIT-PD-L1-TIM3-LAG3-) among the different clinical groups. **C)** Frequency of FAS-expressing CD11c+ DCs among the different clinical groups. **p* < 0.05; Mann Whitney U test. **D)** Linear regression (with 95% interval confidence) showing the association between the frequency of CD8 and FAS-expressing CD11c+ DC. Colors highlight the different clinical groups. IT = Immune Tolerant; IA+ = HBeAg+ Immune Active; IC = Immune Control; IA- = HBeAg- Immune Active; AVT = Anti-Viral Therapy.

**LAG3-TIM3+PD-1+**

[% of Dextr+ CD8 T cells]

**LAG3-TIM3-PD-1+**

[% of Dextr+ CD8 T cells]

**Supplementary Figure 3. A)** Representative flow plots showing expression levels, on HBV-specific CD8 T cells, of other inhibitory (iR) and activating (aR) receptors included in the flow cytometry analysis. Dextramer-positive HBV-specific CD8 T cells (maroon) are overlaid on bulk CD8 T cells (light blue). **B)** Frequency of dextramer-positive HBV-specific samples among the different clinical groups. **C)** Pooled data showing HBV DNA (*left panel*) and HBsAg (*right panel*) levels among patients with [HBVsp(+)] or without [HBVsp(-)] *ex vivo* HBV-specific reactivity (core and/or pool). **D)** Frequency of different iR and aR in bulk, HBV-specific (core and/or pool) and HERPES-specific (CMV and/or EBV) CD8 T cells. *p < 0.05; **p < 0.001; ***p < 0.0001; Wilcoxon signed rank test (bulk vs. Ag-specific) and Mann-Whitney U (HBV-specific vs. HERPES-specific). **E)** Frequency of HBV-specific (core and/or pool) CD8 T cells with phenotype consistent with low exhaustion (LAG3-TIM3-PD-1), intermediate exhaustion (LAG3-TIM3+PD-1+) or high exhaustion (LAG3+TIM3+PD-1+) among the different clinical groups. IT = Immune Tolerant; IA+ = HBeAg+ Immune Active; IC = Immune Control; IA- = HBeAg- Immune Active; AVT = Anti-Viral Therapy.

**Supplementary Figure 4. A)** Pooled data showing the baseline frequency of CD28-expressing and PD-1-expressing CD8 T cells among samples with low or high proliferation levels.

**
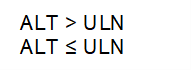

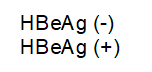

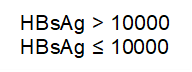

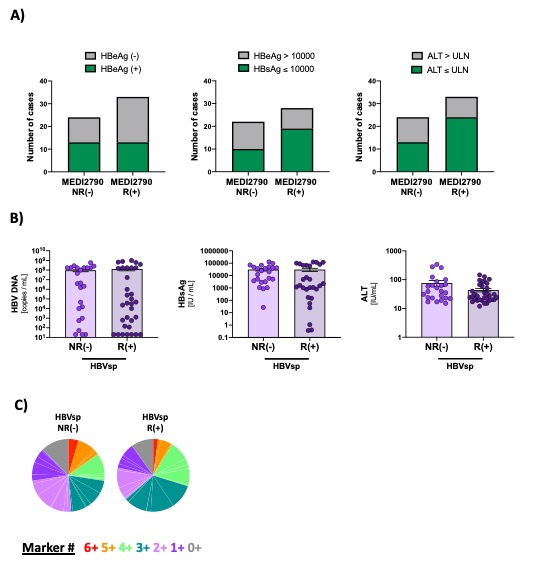
**

**Supplementary Figure 5. A)** Number responders [R(+)] and non-responders [NR(-)] to anti-PD-L1 blockade according to HBeAg seroconversion status (*left panel*), HBsAg levels (*middle panel*) and ALT levels (*right panel*). **B)** Pooled data showing HBV DNA (*left panel*), HBsAg (*middle panel*) and ALT (*right panel*) levels among responders [R(+)] and non-responders [NR(-)] to anti-PD-L1 blockade. **C)** SPICE analysis showing the distribution of marker co-expression (*6+ markers* for CD28+PD-1+TIGIT+PD-L1+TIM3+LAG3+; *0+ markers* for CD28-PD-1-TIGIT-PD-L1-TIM3-LAG3-) among responders [R(+)] and non-responders [NR(-)] to anti-PD-L1 blockade.

**Supplementary Figure 6. A)** Pooled data (n = 6) showing the effect of a mitochondrial antioxidant (MitoQ), IL-12 and MitoQ/IL-12/anti-PD-L1 blockade on the proliferation levels of Ag-specific CD8 T cells. Patients were selected according to sample availability. **B)** Pooled data showing the effect of anti-PD-L1 blockade in TNF (*left panel*), IL-2 (*middle panel*) and IFN$\gamma$+CD107a+ (*right panel*) production. Blue dots show HERPES-specific stimulations (CMV or EBV) while orange dots show HBV-specific stimulations (pool). **C)** Linear regression showing the negative correlation between Ag-specific response and the frequency of highly exhausted (LAG3+TIM3+PD-1+) Ag-specific CD8 T cells. Blue dots show HERPES-specific stimulations (CMV or EBV) while orange dots show HBV-specific stimulations (pool).

**Supplementary Table 1.** Peptide pools.

| Specificity | Source | Description | Peptide composition |
| --- | --- | --- | --- |
| HBV CORE | JPT custom order | 34 HLA class-I restricted T cell epitopes (shared by all genotypes) from the HBV capsid protein and HBV external core antigen | MQLFHLCLIISCSCP CLIISCSCPTVQASK SCPTVQASKLCLGWL ASKLCLGWLWGMDID GWLWGMDIDPYKEFG DIDPYKEFGASVELL EFGASVELLSFLPSD ELLSFLPSDFFPSIR PSDFFPSIRDLLDTA SIRDLLDTASALYRE DTASALYREALESPE YREALESPEHCSPHOH SPEHCSPHHTALRQA PHHTALRQAILCWGE RQAILCWGELMNLAT WGELMNLATWVGSNL LATWVGSNLEDPASR SNLEDPASRELVVSY ASRELVVSYVNVNMG VSYVNVNMGLKIRQL NMGLKIRQLLWFHIS RQLLWFHISCLTFGR HISCLTFGRETVLEY FGRETVLEYLVSFGV LEYLVSFGVWIRTPP FGVWIRTPPAYRPPN TPPAYRPPNAPILST PPNAPILSTLPETTV LSTLPETTVVRRRGR TTVVRRRGRSPRRRT RGRSPRRRTPSPRRR RRTPSPRRRRSQSPR RRRRSQSPRRRRSQS QSPRRRRSQSRESQC |
| HBV POOL | ThinkPeptide | 9 HLA-class-I restricted T cell epitopes from HBV polymerase, envelope, core, core antigen and surface antigen proteins | FLLSLGIHL  FLLTRILTI  FLPSDFFPSI  FLPSDFFPSV  GLSPTVWLSV  WLSLLVPFV  YVNVNMGLK  EYLVSFGVW  KYTSFPWLL |
| PA-CMV-001 | PanaTecs | 5 HLA-class-I restricted T cell epitopes from human cytomegalovirus | NLVPMVATV  SDEEEAIVAYTL  IPSINVHHY  EFFWDANDIY  TPRVTGGGAM |
| PA-EBV-001 | PanaTecs | 15 HLA-class-I restricted T cell epitopes from Epstein Barr Virus | CLGGLLTMV  GLCTLVAML  RVRAYTYSK  RLRAEAQVK  AVFDRKSDAK  IVTDFSVIK  ATIGTAMYK  DYCNVLNKEF  RPPIFIRRL  RAKFKQLL  FLRGRAYGL  QAKWRLQTL  RRIYDLIEL  YPLHEQHGM  EENLLDFVRF |
| PM-ACTS | JPT | Overlapping epitopes of the Actin protein | 15mers with 11aa overlap |
| PM-CEFX-1 | JPT | 176 known peptide epitopes for a broad range of HLA sub-types and different infectious agents for T cell stimulation of populations with a diverse ethnic background | Sequences not available |

**Supplementary Table 2.** Flow cytometry antibodies.

| Specificity | Clone | Fluorophore |
| --- | --- | --- |
| CD8 | LT8 | FITC |
| PD-L1 | 29E.2A3 | BV421 |
| FAS | DX2 | BV510 |
| CD127 | A019D5 | BV570 |
| TIGIT | A15153G | BV605 |
| LAG3 | 11CC65 | BV650 |
| GITR | 108-17 | BV711 |
| CD27 | O323 | BV750 |
| CD14 | M5E2 | BV785 |
| PD-L2 | MIH18 | BUV395 |
| CD16 | 3G8 | BUV496 |
| CD56 | NCAM16.2 | BUV563 |
| CD3 | UCHT1 | BUV661 |
| CD20 | 2H7 | BUV737 |
| CD4 | SK3 | BUV805 |
| ICOS | C398.4A | AF647 |
| 2B4 | C1.7 | AF700 |
| PD-1 | EH12.2H7 | APC Fire 750 |
| TIM-3 | F38-2E2 | PE Dazzle 594 |
| CD28 | CD28.2 | Cy5PE |
| CD11c | 3.9 | Cy55PE |
| 4-1BB | 4B4-1 | Cy7PE |
| CD4 | SK3 | BUV496 |
| CD28 | CD28.2 | BUV563 |
| CD8 | SK1 | BUV805 |
| CD107a | H4A3 | Cy5PE |
| IL-10 | JES3-9D7 | AF488 |
| IFN$\boldsymbol{\gamma}$ | 4S.B3 | BV750 |
| TNF | Mab11 | BV785 |
| IL-2 | MQ1-17H12 | BUV737 |
| GrzB | GRB18 | Cy55PE |

**Supplementary Table 3.** MHCI Dextramers.

| Allele | Peptide | Antigen |
| --- | --- | --- |
| HLA-A*0201 | FLPSDFFPSV | HBV CP |
| HLA-B*3501 | LPSDFFPSV | HBV CP |
| HLA-B*5101 | LPSDFFPSV | HBV CP |
| HLA-A*0201 | WLSLLVPFV | HBV S Protein |
| HLA-A*0201 | GLSPTVWLSV | HBV S Protein |
| HLA-A*0201 | FLLTRILTI | HBV S Protein |
| HLA-A*0201 | NLVPMVATV | CMV pp65 |
| HLA-B*3501 | IPSINVHHY | CMV pp65 |
| HLA-A*0201 | GLCTLVAML | EBV BMLF1 |
| HLA-B*3501 | YPLHEQHGM | EBV EBNA 3A |
